# Support from Social Media during the COVID-19 Pandemic: A Systematic Review

**DOI:** 10.3390/bs14090759

**Published:** 2024-08-28

**Authors:** Stephanie Szeto, Algae Kit Yee Au, Sophie Kai Lam Cheng

**Affiliations:** 1 Felizberta Lo Padilla Tong School of Social Sciences, Saint Francis University, Hong Kong, China; 2 Department of Applied Social Sciences, The Hong Kong Polytechnic University, Hong Kong, China; algae-kit-yee.au@polyu.edu.hk; 3 Department of Psychology, The Chinese University of Hong Kong, Hong Kong, China; klcheng@cuhk.edu.hk

**Keywords:** social media, mental health, social support, emotional support, informational support, COVID-19

## Abstract

The social distancing measures in response to the COVID-19 pandemic have transformed people from social to isolated individuals. During that time, social media became a useful tool for satisfying people’s need for social interaction. Previous systematic reviews, however, have focused largely on the negative impact of social media use and ignored the positive side. Hence, this systematic review examined the role of social media use in providing support—be it social, emotional or informational—during the pandemic. Four databases were systematically searched, and the selection procedure followed the Preferred Reporting Items for Systematic Review and Meta-Analyses (PRISMA) guidelines. Protocol registration: PROSPERO (CRD42022367903). A total of 20 papers were deemed eligible for data extraction. The findings showed that active engagement on social media contributed significantly to maintaining social capital and collective resilience amidst social restrictions. The emotional support obtained from social media was proven effective in alleviating feelings of loneliness and isolation. Also, social media facilitated the rapid dissemination of information and the grassroots mobilization of support by overcoming bureaucratic hurdles and addressing urgent community needs. This review concluded by highlighting the transformative potential of social media in crisis contexts and suggesting implications for mental health interventions and community resilience strategies.

## 1. Introduction and Background

In late 2019, the COVID-19 disease spread across the world after being first reported in Wuhan Province, China. Recognizing the severity of this highly infectious respiratory disease, the World Health Organization (WHO) declared it a global pandemic in early 2020 [1,2]. In response to this health crisis, governments worldwide implemented various measures, including social distancing, mandatory closure of public venues, and suspension of nonessential production and commercial activities [3]. As a result, students transitioned to virtual classes, employees adapted to remote work [4], and social gatherings were harshly restricted [5]. For instance, during the peak of the pandemic, public gatherings of more than two people were prohibited in Hong Kong [6].

While these restrictive measures were crucial in curbing the spread of the virus [7], they also put much of the world’s population into confinement for weeks or even months, profoundly affecting daily life and creating psychological challenges [8]. People of all ages reported feelings of loneliness, anxiety, depression, and social abandonment [9,10,11,12]. To cope with social isolation, they adapted to the “new normal” [13,14] by leveraging existing technologies to seek social [15,16], emotional [12,17], and informational support [18,19] through social media.

### 1.1. The Social Aspect of Social Media

Social media platforms, such as Facebook, Instagram, and Twitter (now known as X), were initially designed to foster social relationships and expand social networks [20]. boyd and Ellison [21] distinguish social media from general websites by emphasizing their social aspect. According to their definition, social media are online social networking platforms which allow users to (i) construct personal profiles so that others recognize the profile owners; (ii) articulate a list of users with whom they have online and/or offline connections, which can be viewed and traversed by others to facilitate social interaction; and (iii) consume, produce, and/or interact with streams of user-generated content that enable information exchange across the online social network. Unlike online forums or communication applications (e.g., WhatsApp, Signal, or Telegram), social media platforms allow users to interact with friends of other users, thereby expanding their social circles.

### 1.2. Stress-Buffering through Social Media

Pre-pandemic research has shown that support from social media could buffer the deleterious impact of stress on mental health [22]. Whether an event is perceived as stressful depends on how people appraise the situation—specifically whether they perceive it as threatening or demanding and feel that it is important to respond but lack appropriate coping mechanisms [23,24]. According to the stress-buffering hypothesis [25], positive and rewarding social interactions provide support that protects people from the potentially adverse effects of stressful events [26]. Support can be categorized into social support, emotional support, and informational support [27]. Social support refers to the extent to which people feel valued and connected within a social network based on communication and reciprocity [28]. Emotional support involves providing care, concern, love, understanding, empathy, and trust [17]. Informational support consists of offering and exchanging advice, recommendations, or knowledge that may help solve problems [29]. Interactions on social media could be instrumental in facilitating these types of support. Social media serve as hubs that connect social networks from various social circles within a unified online space, where user-generated content enables people to express and exchange information [30]. When confronted with stressful situations, people turn to these platforms to disclose and discuss their worries or concerns. The feedback they receive from their social networks fosters a sense of support, contributing to a stress-buffering effect [27]. This effect is essential in helping people adapt, cope, and recover, making social media a means of their coping strategies [31].

### 1.3. Support Obtained through Social Media during COVID-19

During the COVID-19 pandemic, social media platforms emerged as a crucial tool in shaping global adaptation and responses [32]. This importance was underscored by a notable upswing in social media use [33], with Facebook experiencing nearly a 30% traffic surge in early 2020 when the WHO declared the global pandemic [34]. More broadly, there was a 9.9% growth in the number of social media users worldwide [35], indicating the escalating reliance on social media connectivity during this global health crisis [19,36].

This rise in social media use has spurred extensive research, yet findings regarding its positive or negative impacts on mental health are mixed [35,37]. Some studies associate social media use with increased stress and panic, suggesting potential threats to mental well-being [38,39]. For example, the rapid dissemination of frequent updates on death tolls and infection rates, coupled with viral images of people hoarding essentials like toilet paper, could contribute to collective anxiety and drive panic-buying behavior [40]. These examples illustrate the potential negative effects of social media use during the pandemic. Conversely, other research highlights the positive role of social media in providing social, emotional, and informational support during periods of uncertainty and forced social isolation [35]. For instance, people who used social media to maintain social connections reported feeling less isolated [41]. This suggests that the virtual connection on social media could compensate for the lack of face-to-face contact [42]. Therefore, while acknowledging the risks associated with social media use, it is equally important to recognize its benefits for mental well-being.

A number of studies have shown that social support through social media was associated with mitigating the negative impact of COVID-19 on mental health, such as reducing feelings of loneliness and negative mood while fostering social bonding and enhancing social capital [8,15,42,43]. Gilmour et al., [28] indicated that people who used Facebook for social support experienced better mental health outcomes. This suggests that virtual human contacts facilitate social connectedness and help bridge offline social ties during restrictive social distancing measures. When social media was leveraged for meaningful social interaction, it could contribute positively to mental well-being [16].

Emotional support has been shown to have a buffering effect on mental health [44]. Numerous studies highlight that online emotional support could mitigate adverse mental health effects and provide a sense of security. This kind of support allowed people to feel heard, express their feelings, and voice their fears, thereby reducing distress and loneliness [34]. Emotional support is especially important in times of stress and sadness, as it stabilizes recipients’ mental conditions against affective disorders and fosters resilience in the face of mental health challenges [12,45]. During the COVID-19 pandemic, the exchange of emotional support strengthened solidarity and enhanced the collectivist culture, which translated to more effective psychological protection and buffered against the impact of negative emotions [17].

Although the widespread information about the high death tolls and infection rates on social media could heighten feelings of vulnerability and perceived susceptibility of COVID-19 [40], these platforms also serve as valuable sources for those lacking adequate infection prevention control knowledge [13]. The overlapping social circles on social media enable messages to reach beyond traditional methods of information dissemination, such as face-to-face communication and mass media. For instance, infection prevention measures, including hand hygiene and proper way of mask wearing, were widely shared and discussed among social media users [46]. The commenting functions also allowed strangers on social media to exchange experiences and advice, contributing to the availability and accessibility of health information [19]. The reciprocal exchange of information became a tool for offering and seeking support, fostering a sense of solidarity among fellow social media users. The connections formed through these interactions serve as coping tools, enhancing life satisfaction during the uncertainty of the pandemic [18,47].

### 1.4. The Present Study

The social distancing measures confined much of the world’s population for weeks or even months [8]. The prolonged social isolation led to feelings of loneliness and abandonment [10,11,48]. At a time when social contact was largely restricted, social media played a crucial role in helping people stay connected and fostering a sense of support [15,49,50]. These online platforms also enabled quick and efficient social connectivity and information dissemination, providing social, emotional, and informational support during the challenging time [15,49,50].

While some studies indicated that the sharing of rising death tolls, infection rates, and instances of panic-buying on social media could exacerbate stress and panic [38,39,40], social media have also been recognized for their value in building a sense of community [8], enabling and maintaining supportive networks [51], and providing access to infection prevention information [19,46]. The supportive role of social media during the pandemic should not be understated.

Having said that, existing systematic reviews have largely overlooked the specific support that social media provided in terms of fostering social connection. Even though some systematic review papers have attempted to investigate the positive effects of social media use, they often failed to acknowledge the distinct role of social networking due to their focus on general Internet use, a lack of a clear definition of social media, or overgeneralized social media use as part of overall screen time, texting via communication applications, or conflating it with TV viewing [1,14,38,39,50]. Unlike these other forms of screen time and general Internet use, social media enables users to create and interact with friends, acquaintances, and strangers [21]. The collapsed contexts on social media—where lack of spatial, social, and temporal boundaries complicates the maintenance of distinct social circles [30]—combined with the support for user-generated content, facilitate discussion, information generation and transmission across a large user base. Compared to browsing websites, texting, or watching TV, social media offers a richer and wider experience, as users can interact with other familiar and unfamiliar users [21]. Therefore, segregating social media use from general media use may provide more specific insights into how online social networking supported people amid the global public health crisis. Taking these considerations into account, the present systematic review aimed to answer the following research question: how were social media used to facilitate social, emotional, and informational support during the COVID-19 pandemic?

## 2. Materials and Methods

### 2.1. Design

The present systematic review was conducted in accordance with the guidelines of the Preferred Reporting Items for Systematic Reviews and Meta-Analyses (PRISMA) [52,53]. By adopting the integrated mixed methods approach, the empirical studies included quantitative, qualitative, and mixed method designs to capture a greater latitude of the field [54]. The protocol of this systematic review was registered with the International Prospective Register of Systematic Reviews (PROSPERO, protocol number: CRD42022367903) on 17 October 2022.

### 2.2. Inclusion and Exclusion Criteria

Two authors (S.S. and A.K.Y.A.) established the inclusion criteria to determine the eligibility for inclusion in this review. Included articles must: (a) choose the general public (non-clinical human populations) as the sample population; (b) measure the positive effects of social media use on mental well-being (e.g., life satisfaction, alleviating mental health issues, coping with negative emotions); (c) report findings from study designs including randomized-controlled trial (RCT), quantitative nonrandomized (e.g., non-RCTs, case-control studies), quantitative descriptive (e.g., cross-sectional studies, longitudinal studies), qualitative studies (e.g., analysis of social media comments), or mixed methods (i.e., combining quantitative and qualitative methods) in the primary study. English articles published in peer-reviewed journals were selected and subjected to the inclusion criteria [2,38].

Articles were excluded if they: (a) did not meet the inclusion criteria; (b) were not pertinent to COVID-19; (c) mixed the results with non-social media platforms (e.g., general Internet use, general screen time use); (d) used social media as a means of teaching and intervention (e.g., provide education or counselling through social media); (e) recruited clinical samples (e.g., dementia or other mental illnesses); or (f) were letters to editors, commentaries, study protocols, or in a preprint version [2,39].

### 2.3. Search Strategy

Literature searches were conducted in four databases: PsycINFO, PubMed, Scopus, and Web of Science. Two authors (S.S. and S.K.L.C.) performed online searches on 29 October 2022 and an updated search on 1 April 2023 with a date limit from December 2019 (when the first case of COVID-19 was identified [2]) to the date of the search. Across these databases, the key search terms were modified to individual requirements, with Boolean terms used for the following terms and their variations: “COVID-19”, “social media”, “social support”, and “mental well-being”. The full search terms for each database are listed in Table 1.

### 2.4. Screening and Data Extraction

Two authors (S.S. and A.K.Y.A.) initially screened the titles and abstracts of the identified articles to determine their eligibility. Then, all three authors independently reviewed the full text of selected articles. Any discrepancies in eligibility were discussed until agreement was reached.

Data from all included full-text articles were extracted to obtain the following information: (a) study aims; (b) study designs (e.g., cross-sectional, qualitative, mixed methods); (c) data collection periods; (d) sample characteristics, including sample size, age, and population; (e) measurements; and (f) study outcomes. The study designs, data collection periods, sample characteristics, and study outcomes were considered relevant variables that could facilitate the classification and comparison of the selected articles. Such data were compiled into a standardized spreadsheet.

To examine the interrater reliability, Fleiss’ Kappa was employed to statistically compare the ratings across three authors [55]. The average of Fleiss’ Kappa was 0.86 and the confidence interval was between 0.86 and 0.87, indicating a high level of rater agreement [56,57].

**Figure 1:**
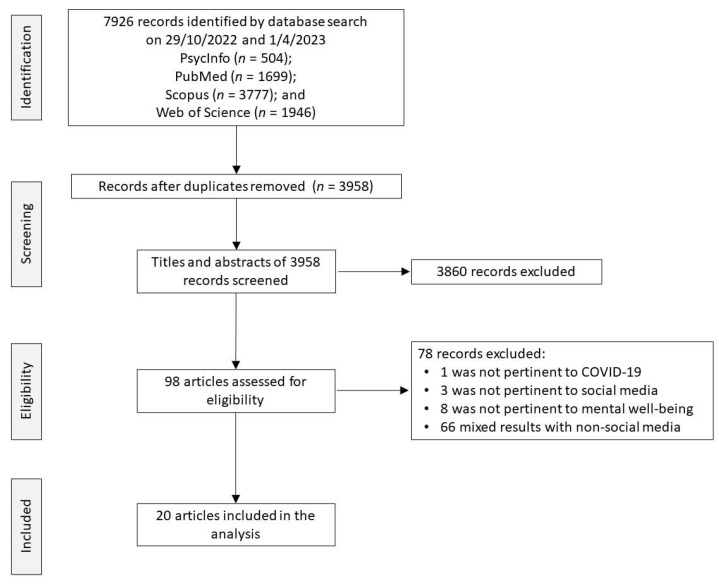
PRISMA Flow diagram.

The PRISMA selection flow chart [53] is presented in Figure 1 to outline the selection process. Where studies were excluded for not fulfilling the inclusion criteria, or for meeting the exclusion criteria, detailed reasons are indicated in the flowchart.

### 2.5. Study Quality and Risk-of-Bias Assessment

The Mixed Methods Assessment Tool (MMAT) is a quality assessment protocol designed specifically for systematic reviews of articles with qualitative, quantitative, and mixed method designs [58]. This critical appraisal tool includes screening questions to eliminate non-empirical studies from the appraisal stage. Then, five categorical questions (yes, no, and cannot tell) are structured to determine the study design: (a) qualitative, (b) randomized controlled, (c) nonrandomized, (d) quantitative descriptive, and (e) mixed methods. Questions rated yes are counted toward the overall score, and the total score for each research article ranges from 1 (receive one yes) to 5 (all five questions are rated yes) [59]. The MMAT has high reliability and efficiency as a quality assessment protocol and can concurrently appraise methodological quality across various empirical research [2]. This tool was used to appraise the methodological quality of the selected articles in the present systematic review. Three authors independently conducted the appraisal with it to minimize selective perception and interpretive bias.

### 2.6. Data Synthesis

All three authors contributed to data synthesis. Since the selected studies had great heterogeneity in measuring and reporting outcomes, a narrative approach to synthesizing the findings was deemed appropriate [54].

## 3. Results

The searches identified 7926 articles, of which 3958 articles remained after removing duplicates. A total of 3860 articles were excluded after the first screening of the title and abstract analysis. The remaining 98 articles were subjected to a full-text evaluation, and 78 of them were excluded for the following reasons: one was not pertinent to COVID-19; three were not pertinent to social media platforms; eight were not pertinent to promoting mental well-being; and 66 had results mixed with non-social media. Finally, 20 articles were included in this review [8,9,10,12,15,18,34,47,49,51,60,61,62,63,64,65,66,67,68,69]. A description of these selected articles is provided in Table 2.

### 3.1. Study Designs and Data Collection Periods

Among the 20 selected articles, 15 of them employed a cross-sectional design [9,12,15,18,34,47,49,60,61,64,65,66,67,68,69], two employed a qualitative design [10,63], and one was an experimental study [8]. Five papers focused on one single social media platform, either Facebook (four) [10,49,51,66] or Instagram (one) [64]. The remaining 15 articles centered around multiple social media platforms [8,9,12,15,18,34,47,60,61,62,63,65,67,68,69]. Fourteen articles reported data collected in 2020 [8,9,12,15,18,34,47,49,51,60,61,62,65,69], three in 2021 [66,67,68], and one in 2022 [64]. The remaining two articles did not specify the data collection period [10,63].

### 3.2. Participant Characteristics

The number of participants ranged from 215 [61] to 3474 [65] for the cross-sectional studies. The two qualitative studies recruited 40 [10] and 19 [63] participants respectively. Two other articles employed a mixed methods approach, with 307 [62] and 181 [51] participants, respectively. The remaining experimental paper recruited 681 participants [8].

Fifteen articles recruited adults who used social media during the COVID-19 pandemic. Eight of these articles focused on specific social media platforms [8,9,10,49,51,64,66,69], while seven articles did not specify the social media platforms under investigation [18,34,47,60,63,65,67]. Two articles recruited adolescents [12,68] and the other two recruited college students [15,61]. The remaining article recruited both college students and general social media users [62].

Sixteen articles recruited participants from the same region [9,10,12,15,18,34,47,51,60,61,62,63,64,66,68,69], three included participants from different regions [8,65,67], and one recruited participants from Amazon Mechanical Turk (MTurk) without specifying the targeted region [49].

### 3.3. Quality Assessment Outcomes

The quality of the selected articles was assessed using the MMAT scoring system [58,59]. These articles were assigned scores from 1 (the lowest) to 5 (the highest). Four articles (20 percent) were rated the highest score of 5; indicating their high quality. Eleven articles (55 percent) were rated 4, two articles (10 percent) were rated 3, one article (five percent) was rated 2, and the remaining two articles (10 percent) that employed a mixed methods approach were rated the lowest score of 1. The low-quality ratings were attributed to the inadequate integration of quantitative and qualitative findings within the mixed methods research design. The MMAT ratings are presented in Table 2.

### 3.4. Study Outcomes

#### 3.4.1. Social Support

Four articles focused on the social benefits of using social media platforms during COVID-induced social isolation [8,9,15,69]. Two of them found that spending time on social media was associated with greater feelings of social connection and higher social support [15] and predicted a greater sense of community and maintenance of social capital [9]. One article revealed that active social media use was positively associated with social support [69]. The remaining one was an experimental study which supported the findings of the former papers, suggesting that spending time browsing social media was associated with greater feelings of social connectedness [8]. These findings indicated that social media platforms were valuable in fostering social support and sustaining connections during the pandemic.

#### 3.4.2. Emotional Support

The majority of the selected articles (65%) examined emotional support from social media use [10,12,34,49,60,61,62,63,64,65,66,67,68]. One qualitative study found that uploading photographs of late family members on Facebook facilitated the working-through of bereavement [10]. Four articles revealed that participants used social media as a means to cope with stress [49,61] and negative emotions [12,63]. Eight articles investigated the relationship between social media use and well-being. It was found that social media use was positively linked to satisfaction with life [67] and mental well-being [60,64,65,66,68] while negatively associated with feelings of loneliness [62] and isolation [34]. Overall, these findings highlighted the importance of social media use in supporting people’s coping strategies and emotional well-being during the COVID-19 pandemic.

#### 3.4.3. Informational Support

Three articles found that social media use was related to informational support [18,47,51]. Two of them showed that reciprocal sharing of COVID-19-related information provided a sense of support that increased life satisfaction [18,47]. The remaining article found that social media platforms facilitated the sharing of credible advice, government press releases, and community resources such as information on grocery donations [51]. These findings emphasized the valuable role of social media in providing informational support and access to relevant resources during the pandemic.

## 4. Discussion

The systematic review aimed to add to the knowledge by exploring and synthesizing existing empirical evidence on the effect of social media use on social, emotional, and informational support during the COVID-19 pandemic. Online searches in four databases were performed and 20 papers were selected for data extraction after the screening process. Their methodologies were greatly heterogenous, ranging from quantitative, qualitative, and mixed method designs. Hence, a narrative approach was employed for the synthesis of findings [54]. 15 papers (75 percent) were assessed to have high quality (MMAT quality scores of 4 and 5), and the remaining 5 papers (25 percent) were rated moderate to low quality.

The pandemic created an unprecedented global impact. During the lockdown, prolonged social isolation became a major cause of stress and anxiety worldwide [70]. Against this backdrop, social media emerged as a powerful tool for people to rebuild social ties and connect with long-distance family and friends [62]. Relationship reconnection on social media has shown its value during various crises in previous research [71]. In times when people feel powerless due to offline catastrophes, they proactively utilize social media platforms to strengthen online social capital through connecting and reconnecting with their loved ones [8].

### 4.1. Social Support Facilitated by Active Use of Social Media

A number of studies have found that active use of social media had a positive association with well-being during the COVID-19 pandemic. This included actively connecting with family and friends, seeking social support, and sharing life experiences. However, such association was not found in passive social media users, such as people who passively scrolled through posts [12,60,62].

During quarantine and social distancing measures, active social media users who shared personal feelings and life experiences would have a higher likelihood of receiving positive feedback on their online networks. Through interaction with other users, they were more likely to foster a stronger sense of social support that helped them better manage their feelings of loneliness and isolation [65]. These findings were supported by an experimental study, in which a group of participants were asked to spend 10 min using social media actively while their counterparts were instructed not to use social media. The result showed that those who actively used social media reported greater feelings of social connection and lower feelings of social isolation during social distancing measures [8,15]. It seems to be conclusive that active use of social media increased social connection, which in turn facilitated social support when in-person contacts were inhibited by social restrictive measures [72].

These findings during the pandemic aligned with those of pre-pandemic research, indicating that active use of social media could have a positive effect on well-being compared to passive use of these platforms [49,69]. Social media provided people with an accessible and cost-effective means of seeking social support when facing daily stress and mental health issues. Taking Facebook as an example, active users received higher levels of social support [24,73]. The interaction with family and friends, such as liking, sharing, and commenting on their posts, encouraged disclosure and discussion about stressful events or depressive symptoms [22,74]. According to the stress-buffering hypothesis [25], receiving assistance from others is a coping strategy that helps reduce stress levels and improve mental well-being [27]. During the pandemic, active use of social media enacted social support that created a stress-buffering effect [4,61]. When expressing feelings or sharing problems, this buffering effect could be attributed to two mechanisms. First, venting negative emotions unburdened oneself and created a sense of relief. Hence, adverse events and negative feelings became less toxic through the “cathartic effect” ([75] p. 263). Second, self-disclosure allowed people to take a break from thinking about upsetting events. They could then reevaluate the problem and sort out their memory, so as to enhance their understanding of the events and themselves [76]. On the other hand, users passively scrolling through posts after posts were less likely to receive supportive responses. Therefore, they might not be able to gain the psychological benefits [77].

Based on the research conducted before and during the COVID-19 pandemic, active engagement in social media posts fostered a sense of social support, which created a stress-buffering effect to enhance mental well-being, in contrast to those who passively read posts and had limited interaction with others [4,24,27,61].

### 4.2. Negative Emotions Relieved through Community Building on Social Media

Traditional community resilience relies on a well-networked community to cope with public emergencies [78]. However, COVID-induced quarantines and lockdowns hindered in-person interactions, thereby posing challenges to community resilience. During the mandatory quarantine period, people reported experiencing mental ill-health, such as emotional distress, depression, and suicidal ideation [79,80,81]. To maintain a sense of community identity in terms of belongingness and relatedness [78], community members shifted their traditional offline in-person networking activities to online platforms. As they fostered their collective community identity on social media, they facilitated the development of social capital, nurtured a sense of belonging, and enhanced the perceived community resilience, which ultimately helped them cope with emotional challenges [9]. By mitigating feelings of isolation, social media played an integral role in cultivating a positive psychological sense of community [15] and promoting mental well-being among community members [34].

Since social distancing measures restricted visits to nursing homes, many people were forced to separate from their critically ill family members. Some of them could not say a final goodbye to their loved ones and suffered from self-blame [82]. In this regard, social media platforms became the conduits for expressing intense feelings of frustration and guilt. For example, one study found that people created Facebook Groups dedicated to COVID-19 grievers [10]. Online funeral ceremonies were organized for those who died of the pandemic and the virtual collective mourning, whether religious or not, unified the bereaved to work through the grief as a way to relieve negative emotion [83]. In some Facebook groups, members uploaded photos, shared stories, and bid farewell to the deceased to voice their pain and loss [10]. Group members who were initially strangers became Facebook friends and formed a supportive community where they shared memories and processed grief through online interaction, such as liking, sharing, and commenting on posts. When face-to-face bereavement was not possible, virtual reciprocal emotional support helped relieve frustration and guilt. It also enhanced positive effect and promoted a sense of solidarity [10,62]. Thus, these Facebook Groups empowered people to organize digital funerals, providing recognition and restoring human identity.

People who turned to social media as a recollection of past events and interacted regularly reported feeling less isolated [49] and higher life satisfaction [67]. When face-to-face interaction was restricted, these virtual relational connections were integral to providing crucial emotional support to reduce feelings of loneliness and emotional exhaustion [61,62,63,64]. Through caring and encouragement [68], social media enabled people to cope with the significant stress and uncertainty they faced offline [84].

### 4.3. Information Dissemination on Social Media

The closure of care homes during the COVID-19 pandemic left many care recipients deprived of essential support and services. In this context, informational support provided through social media was found to be pivotal for caregivers under lockdown [18]. A study found that “care-mongering” groups were created on Facebook—a social media-driven initiative that mobilized community support through effective information dissemination to aid vulnerable people [13]. These groups facilitated community-led information exchange on crucial matters such as grocery donations, personal protective equipment (PPE) distribution, and health-related issues, all of which were vital to the disadvantaged population [51].

While some previous studies have indicated the potential negative effect of rapid information dissemination on social media—such as spread of distressing news about rising death tolls, infection rates, and images of panic-buying, which could create negative emotion contagion [38,39,40]—other research showed the positive impact of sharing useful and constructive information. Social media users who exchanged positive information about COVID-19 reported higher levels of life satisfaction, a sense of adequacy, and reduced levels of depression and anxiety [47]. The unbeatable uncertainty surrounding the infectious disease posed challenges and obstacles, yet amidst such adversity, people shared information to provide general advice about disease prevention, thereby helping alleviate anxiety and increasing life satisfaction [18].

Furthermore, information exchange on social media was found to be positively related to informational reciprocity, fostering collective efforts to sustain the support for caregivers. Offering informational support on social media predicted coping behaviors, such as allocations of donated food, personal hygiene materials, and other resources [51]. In this regard, social media platforms became fundamental for the rapid and extensive sharing of opinions, ideas, and information, serving as valuable sources of knowledge related to COVID-19 and caregiving support [66].

### 4.4. Contributions, Implication, and Limitations

This paper contributed to the literature by providing a systematic review and synthesis of empirical evidence on how social media became essential tools for various forms of social, emotional, and informational support during the COVID-19 pandemic. Previous studies showed that active use of social media, including self-disclosure of personal feelings and life experiences, created a stress-buffering effect [4,61]. This engagement in social media use helped alleviate negative feelings of loneliness and isolation that arose from restricted interpersonal contact in the pandemic [65]. Additionally, information sharing on social media facilitated allocation of donated resources among community members, particularly when access to grocery stores was restricted and shortage of personal hygiene materials arose due to government expropriations [5].

The findings from the present systematic review also provided implications for future research directions in the area of mental health interventions. Social media users were found to develop virtual communities that helped maintain a sense of community identity and mutual support, thereby mitigating feeling of isolation [9,15,34]. Further research may explore the possibility of training volunteers to provide peer-based social, emotional, and informational support via social media platforms. This approach could strengthen social capital, enhance solidarity, and improve community resilience, preparing communities for future infections crises [49].

While previous literature has criticized large bureaucratic organizations for being slow, ineffective, and rigid in offering timely assistance [85], the present review highlighted how social media platforms could provide an agile and decentralized information dissemination and support [86]. These platforms enabled users to flexibly and creatively allocate resources within their communities without the need to navigate bureaucratic hassle [51]. This suggests practical implications for bottom-up informational support in future unpredictable pandemics.

However, social media could be a double-edged sword. While studies have shown that information disseminated through social media helped people feel more prepared to handle the novel virus [14], concerns have also been raised about its use. The rapid transmission of information on social media, particularly during periods of uncertainty, could lead to the spread of misinformation. For instance, misinformation about wearing space suit-like protective costumes [87] and drinking herbal tea as a COVID-19 treatment [88] could undermine proper infection prevention measures and delay appropriate treatment. The overall impact of social media—whether these platforms offer more benefits or risks—remains inconclusive. Further research is needed to identify the factors and circumstances that determine if social media use has positive or negative influences on mental well-being.

This systematic review has several limitations. First, the inclusion of studies published in English only may limit the diversity of the reviewed literature, potentially overlooking valuable research conducted in other languages. Second, this review did not examine the actual and perceived social support on social media. Actual social support is the support that was actually received during the pandemic, such as recommendations or hygiene materials. On the other hand, perceived social support is the belief that one would receive support when they sought on social media [35]. As previous research has found both actual and perceived social support positively contributed to collective resilience to overcome public health crises [89], further review may investigate how these two types of support enhanced well-being during the pandemic. Third, the present review focused only on the COVID-19 pandemic. A review of social media use in various public health crises would help generate valuable knowledge about the contribution of advanced internet technology to mental well-being when face-to-face interactions are restricted in a more general context.

## 5. Conclusions

This systematic review highlighted the significant role of social media in providing social, emotional, and informational support during the COVID-19 pandemic. The findings revealed that the active use of social media platforms, such as engaging in meaningful interactions and sharing personal experiences, was associated with improved mental well-being as it fostered social connections and provided a buffer against feelings of loneliness and isolation. The formation of online communities also helped mitigate negative emotions, offering a sense of belonging and collective resilience during a period of widespread social restrictions. Furthermore, the review underscored the importance of social media in disseminating crucial information and mobilizing community-driven support, thereby enhancing collective efforts to address public health challenges faced by vulnerable populations. In terms of contribution to literature and practical knowledge, this review suggested that social media could facilitate bottom-up resources allocation and avoid bureaucratic delays, which proved invaluable during the crisis. In conclusion, social media emerged as a vital tool for maintaining social support, emotional well-being, and information exchange during the COVID-19 pandemic. Its potential in promoting peer-based support and enhancing community resilience points to valuable applications for future mental health interventions and crisis management strategies.

## Figures and Tables

**Table 1:** Databases and search terms.

Databases	Search Terms	29 October 2022	1 April 2023
PsycINFO	tiab(COVID-19 OR “COVID 19” OR covid* OR coronavirus* OR “novel coronavirus” OR “new coronavirus” OR quarant* OR pandemic OR 2019-nCoV OR SARS-CoV-2 OR lockdown OR “lock down” OR “social isolation” OR confinement) AND tiab(“social media” OR “social networking” OR “social networking site” OR “SNS” OR “online social networking” OR “social media use” OR “social media exposure” OR “Facebook” OR “Twitter” OR “Instagram” OR “Weibo”) AND tiab(“psychological well-being” OR “psychological wellbeing” OR “psychological well being” OR “psychological health” OR “mental health” OR “mental well-being” OR “mental wellbeing” OR “mental well being” OR “subjective well-being” OR “subjective wellbeing” OR “subjective well being” OR “social well-being” OR “social wellbeing” OR “social well being” OR “emotional well-being” OR “ emotional wellbeing” OR “ emotional well being” OR “life satisfaction” OR “positive mood” OR cope OR coping OR resilien* OR “peer support” OR “social support” OR “social connection” OR “social capital”)	426	78
PubMed	((COVID-19[Title/Abstract] OR “COVID 19”[Title/Abstract] OR covid*[Title/Abstract] OR coronavirus*[Title/Abstract] OR “novel coronavirus”[Title/Abstract] OR “new coronavirus”[Title/Abstract] OR quarant*[Title/Abstract] OR pandemic[Title/Abstract] OR 2019-nCoV[Title/Abstract] OR SARS-CoV-2[Title/Abstract] OR lockdown[Title/Abstract] OR “lock down”[Title/Abstract] OR “social isolation”[Title/Abstract] OR confinement[Title/Abstract]) AND (“social media”[Title/Abstract] OR “social networking”[Title/Abstract] OR “social networking site”[Title/Abstract] OR “SNS”[Title/Abstract] OR “online social networking”[Title/Abstract] OR “social media use”[Title/Abstract] OR “social media exposure”[Title/Abstract] OR “Facebook”[Title/Abstract] OR “Twitter”[Title/Abstract] OR “Instagram”[Title/Abstract] OR “Weibo”[Title/Abstract])) AND (“psychological well-being”[Title/Abstract] OR “psychological wellbeing”[Title/Abstract] OR “psychological well being”[Title/Abstract] OR “psychological health”[Title/Abstract] OR “mental health”[Title/Abstract] OR “mental well-being”[Title/Abstract] OR “mental wellbeing”[Title/Abstract] OR “mental well being”[Title/Abstract] OR “subjective well-being”[Title/Abstract] OR “subjective wellbeing”[Title/Abstract] OR “subjective well being”[Title/Abstract] OR “social well-being”[Title/Abstract] OR “social wellbeing”[Title/Abstract] OR “social well being”[Title/Abstract] OR “emotional well-being”[Title/Abstract] OR “ emotional wellbeing”[Title/Abstract] OR “ emotional well being”[Title/Abstract] OR “life satisfaction”[Title/Abstract] OR “positive mood”[Title/Abstract] OR cope[Title/Abstract] OR coping[Title/Abstract] OR resilien*[Title/Abstract] OR “peer support”[Title/Abstract] OR “social support”[Title/Abstract] OR “social connection”[Title/Abstract] OR “social capital”[Title/Abstract])	1358	341
Scopus	(TITLE-ABS-KEY (COVID-19 OR “COVID 19” OR covid* OR coronavirus* OR “novel coronavirus” OR “new coronavirus” OR quarant* OR pandemic OR 2019-ncov OR SARS-CoV-2 OR lockdown OR “lock down” OR “social isolation” OR confinement) AND TITLE-ABS-KEY (“social media” OR “social networking” OR “social networking site” OR “SNS” OR “online social networking” OR “social media use” OR “social media exposure” OR “Facebook” OR “Twitter” OR “Instagram” OR “Weibo”) AND TITLE-ABS-KEY (“psychological well-being” OR “psychological wellbeing” OR “psychological well being” OR “psychological health” OR “mental health” OR “mental well-being” OR “mental wellbeing” OR “mental well being” OR “subjective well-being” OR “subjective wellbeing” OR “subjective well being” OR “social well-being” OR “social wellbeing” OR “social well being” OR “emotional well-being” OR “ emotional wellbeing” OR “ emotional well being” OR “life satisfaction” OR “positive mood” OR cope OR coping OR resilien* OR “peer support” OR “social support” OR “social connection” OR “social capital”)) AND PUBYEAR > 2018 AND PUBYEAR < 2023 AND (LIMIT-TO (LANGUAGE, “English”))	3023	754
Web of Science	Search titles: ((TI = (COVID-19 OR “COVID 19” OR covid* OR coronavirus* OR “novel coronavirus” OR “new coronavirus” OR quarant* OR pandemic OR 2019-nCoV OR SARS-CoV-2 OR lockdown OR “lock down” OR “social isolation” OR confinement)) AND TI = (“social media” OR “social networking” OR “social networking site” OR “SNS” OR “online social networking” OR “social media use” OR “social media exposure” OR “Facebook” OR “Twitter” OR “Instagram” OR “Weibo”)) AND TI = (“psychological well-being” OR “psychological wellbeing” OR “psychological well being” OR “psychological health” OR “mental health” OR “mental well-being” OR “mental wellbeing” OR “mental well being” OR “subjective well-being” OR “subjective wellbeing” OR “subjective well being” OR “social well-being” OR “social wellbeing” OR “social well being” OR “emotional well-being” OR “ emotional wellbeing” OR “ emotional well being” OR “life satisfaction” OR “positive mood” OR cope OR coping OR resilien* OR “peer support” OR “social support” OR “social connection” OR “social capital”) Search abstracts: ((AB = (COVID-19 OR “COVID 19” OR covid* OR coronavirus* OR “novel coronavirus” OR “new coronavirus” OR quarant* OR pandemic OR 2019-nCoV OR SARS-CoV-2 OR lockdown OR “lock down” OR “social isolation” OR confinement)) AND AB = (“social media” OR “social networking” OR “social networking site” OR “SNS” OR “online social networking” OR “social media use” OR “social media exposure” OR “Facebook” OR “Twitter” OR “Instagram” OR “Weibo”)) AND AB = (“psychological well-being” OR “psychological wellbeing” OR “psychological well being” OR “psychological health” OR “mental health” OR “mental well-being” OR “mental wellbeing” OR “mental well being” OR “subjective well-being” OR “subjective wellbeing” OR “subjective well being” OR “social well-being” OR “social wellbeing” OR “social well being” OR “emotional well-being” OR “ emotional wellbeing” OR “ emotional well being” OR “life satisfaction” OR “positive mood” OR cope OR coping OR resilien* OR “peer support” OR “social support” OR “social connection” OR “social capital”)	1568	378
	Total search outcome:	7926	

**Table 2:** Methodological features and quality appraisal of included studies.

Authors	Study Designs	Social Media Platforms	Date of Data Collection and Regions	Sampling	Key Findings	Quality Appraisal
Bishop et al., 2022 [51]	Mixed method	Facebook	Qualitative: Fall 2020 to Spring 2021; quantitative: September to November 2020; in Canada	181 members of care-mongering Facebook Groups (qualitative: 16 and quantitative: 165 Age range: from under 35 to over 80	Informational support: Information exchange on care-mongering Facebook Groups facilitated, for example, donation distribution.	1
Cauberghe et al., 2021 [12]	Cross-sectional	General social media platforms	16 to 30 April 2020 in Belgium	2165 adolescents Mean age: 15.51	Emotional support: Using social media as an active coping mediated the relationships between anxiety and happiness.	5
Chuang, 2022 [66]	Cross-sectional	Facebook	25 May to 7 June 2021 in Taiwan	340 users Age range: from under 20 to above 41	Emotional support: Social media use was positively associated with subjective well-being.	4
Feng et al., 2022 [63]	Qualitative	General social media platforms	In China without specifying period	19 users Age range: from 20 to 67	Emotional support: Communication with others on social media helped relieve psychological pain, anxiety, and depression.	4
Helm et al., 2022 [34]	Cross-sectional	General social media platforms	Study 1: 26 March 2020; Study 2: 2 to 24 April 2020 in United States	1446 users (Study 1: 299; Study 2: 1147) Mean age: Study 1 = 38.75; Study 2 = 37.47	Emotional support: Study 1: social media use predicted lower social loneliness. Study 2: active social media use predicted meaning in life through lower social loneliness.	4
Lee-Won et al., 2023 [67]	Cross-sectional	General social media platforms	Study 1: June 2021 in United States; study 2: October 2021 in South Korea	1995 users (Study 1: 485; Study 2: 1510) Mean age: Study 1 = 43.17; Study 2 = 47.68	Emotional support: Recollection of one’s past events on social media was positively associated with life satisfaction.	4
Liu et al., 2023 [18]	Cross-sectional	General social media platforms	February 2020 in China	511 users Mean age: 32.57	Informational support: Information sharing on social media was positively related to informational reciprocity and life satisfaction.	4
Maftei et al., 2023 [68]	Cross-sectional	General social media platforms	23 September to 7 October 2021 in Romania	258 adolescents Mean age: 13.38	Emotional support: Social media use was positively associated with well-being.	2
Mantymaki et al., 2022 [49]	Cross-sectional	Facebook	Early May 2020 from MTurk without specifying nations	398 users Age range: from 20 to 69	Emotional support: Using social media to detach from COVID-19 stressors had a positive effect on mental well-being.	3
Masciantonio et al., 2021 [69]	Cross-sectional	Facebook, Twitter, Instagram, and TikTok	7 to 16 April 2020 in Belgium	793 users Mean age: 33.75	Social support: On Facebook: passive use of social media was negatively associated with satisfaction with life. On Twitter: active use of social media was positively associated with satisfaction with life through social support. On Instagram: active use of social media was positively associated with satisfaction with life through social support. On TikTok: both active and passive use showed no significant association with satisfaction with life.	4
Midgley et al., 2022 [8]	Experimental	Facebook and Instagram	27 April to 8 May 2020 in Canada and United States	681 users Mean age: 35.58	Social support: Spending time on social media was associated with greater feelings of social connection.	4
Pennington, 2021 [62]	Mixed method	Facebook, Twitter, and Instagram	Mid-April 2020 in United States	307 college students and general social media users Mean age: 34.22	Emotional support: Quantitative: Active use of social media was associated with lower loneliness. Qualitative: social media facilitated connection with long-distance family and friends, and previously lapsed social ties.	1
Sitar-Taut et al., 2021 [15]	Cross-sectional	General social media platforms	May 2020 in Romania	544 college students Age: 55.33% under 22 years of age	Social support: Social media use predicted bridging and maintaining social capital.	5
Staniewski & Awruk, 2022 [64]	Cross-sectional	Instagram	January 2022 in Poland	359 users Age range: from 25 to over 49	Emotional support: Majority of the respondents (71%) did not consider using Instagram would have negative impact on their mental well-being, as opposed to 8% reported worsened mental well-being as a result of using Instagram.	4
Testoni et al., 2021 [10]	Qualitative	Facebook	In Italy without specifying period	40 grievers on Facebook Mean age: 47	Emotional support: Work-through of bereavement on Facebook Groups dedicated to the COVID-19 grievers	4
Thygesen et al., 2022 [65]	Cross-sectional	General social media platforms	24 October to 29 November 2020 in United States, UK, Norway, and Australia	3474 users Aged 18 or above	Emotional support: Mental well-being was positively associated with motives for contacting others and maintaining relationships.	5
Xie et al., 2022 [9]	Cross-sectional	Facebook and Instagram	October 2020 in United States	676 users Mean age: 41.1	Social support: Using social media for seeking social support was positively associated with community resilience.	5
C C Yang et al., 2020 [60]	Cross-sectional	General social media platforms	May 2020 recruited Asians in United States	242 users Mean age: 32.88	Emotional support: Active use of social media predicted subjective well-being.	4
Y Yang et al., 2020 [47]	Cross-sectional	General social media platforms	19 to 21 February 2020 in China	3159 users Aged 18 or above	Informational support: Information sharing on social media was positively related to sense of adequacy, whereas that is negatively related to anxiety.	4
Zhen, 2021 [61]	Cross-sectional	General social media platforms	8 to 25 April 2020 in United States	215 college students Mean age: 20.5	Emotional support: Disclosure of personal issues to friends on social media helped reduce perceived stress caused by COVID-19.	3

## Data Availability

Data sharing is not applicable to this article—no new data was created or collected in this study.
